# Identification of a Disulfide Bridge Important for Transport Function of SNAT4 Neutral Amino Acid Transporter

**DOI:** 10.1371/journal.pone.0056792

**Published:** 2013-02-22

**Authors:** Rugmani Padmanabhan Iyer, Sumin Gu, Bruce J. Nicholson, Jean X. Jiang

**Affiliations:** Department of Biochemistry, University of Texas Health Science Center, San Antonio, Texas, United States of America; University of Bern, Switzerland

## Abstract

SNAT4 is a member of system N/A amino acid transport family that primarily expresses in liver and muscles and mediates the transport of L-alanine. However, little is known about the structure and function of the SNAT family of transporters. In this study, we showed a dose-dependent inhibition in transporter activity of SNAT4 with the treatment of reducing agents, dithiothreitol (DTT) and Tris(2-carboxyethyl)phosphine (TCEP), indicating the possible involvement of disulfide bridge(s). Mutation of residue Cys-232, and the two highly conserved residues Cys-249 and Cys-321, compromised the transport function of SNAT4. However, this reduction was not caused by the decrease of SNAT4 on the cell surface since the cysteine-null mutant generated by replacing all five cysteines with alanine was equally capable of being expressed on the cell surface as wild-type SNAT4. Interestingly, by retaining two cysteine residues, 249 and 321, a significant level of L-alanine uptake was restored, indicating the possible formation of disulfide bond between these two conserved residues. Biotinylation crosslinking of free thiol groups with MTSEA-biotin provided direct evidence for the existence of a disulfide bridge between Cys-249 and Cys-321. Moreover, in the presence of DTT or TCEP, transport activity of the mutant retaining Cys-249 and Cys-321 was reduced in a dose-dependent manner and this reduction is gradually recovered with increased concentration of H_2_O_2_. Disruption of the disulfide bridge also decreased the transport of L-arginine, but to a lesser degree than that of L-alanine. Together, these results suggest that cysteine residues 249 and 321 form a disulfide bridge, which plays an important role in substrate transport but has no effect on trafficking of SNAT4 to the cell surface.

## Introduction

Amino acid transporters play essential roles in the uptake of nutrients, protein synthesis, chemical metabolism, detoxification, and neurotransmitter cycling [1]. Sodium-coupled neutral amino acid transporters (SNAT), also known as the solute carrier 38 (SLC38) transporters, belong to amino acid/auxin permease (AAAP) gene family of anion-polyamine-organocation (APC) superfamily [2], [3]. These are sodium and pH-dependent transporters that mainly mediate the transport of neutral amino acids essential for cellular functions [4]. Six members of the SNAT family of transporters are characterized. These transporters are divided into two subfamilies – system A and system N. Members of systems A subfamily mainly transport amino acid with aliphatic side chains, including SNAT1 (SLC38A1), SNAT2 (SLC38A2) and SNAT4 (SLC38A4). On the other hand, system N transporters transport amino acids with nitrogen in their side chain consisting of SNAT3 (SLC38A3), SNAT5 (SLC38A5) and SNAT7 (SLC38A7) [4], [5]. SNAT4 exhibits functional and regulatory properties of classically defined system A transporters [6]. This transporter contains 547 amino acid residues with a predicted molecular mass of 55 kDa. SNAT4 predominantly transports L-alanine followed by L-histidine and L-glutamine [6]. Interestingly, SNAT4 is also suggested to transport cationic amino acids independent of sodium gradient [7]. SNAT4 is primarily expressed in liver, muscle and placenta [6], [8]–[11]. SNAT4 is reported to be functional in the first trimester placenta microvillous membrane, but has minimal contributions at term. A previous study from our laboratory has shown that SNAT4 plays a crucial role in liver physiology *via* PI3-kinase signaling pathway [8], [11]. Despite the physiological importance of SNAT4 in mammalian physiology, relatively little is known about the structure and function of these transporters. Our recent topological study showed that SNAT4 consists of ten transmembrane segments with both N and C termini facing the extracellular side [12]. However, the exact three-dimensional structure and important structural motifs and residues involved in the transport function of SNAT family of transporters are still unknown. Better understanding of the structural information is essential for delineating the mechanism of transport associated with this class of transporters.

Disulfide bonds formed by cysteine residues have been found to play roles in various transporter proteins, including protein intracellular trafficking [13], [14], delivery to cell surface [13]–[15], protein oligomerization [16], [17] and substrate transport function [18], [19]. In addition, the unique chemistry of cysteine has made it useful in various enzymatically active sites [20]–[22]. In this study, we identified a disulfide bridge formed by cysteine residues 249 and 321, which plays an important role in substrate transport by SNAT4, but has no effect on trafficking of SNAT4 to the cell surface.

## Materials and Methods

### Materials

Quick Change Site Directed Mutagenesis Kit™ was purchased from Stratagene (La Jolla, CA). Leibovitz (L-15) medium, dithiothreitol (DTT), Tris(2-carboxyethyl)phosphine hydrochloride solution (TCEP), glutathione (GSH), Penicillin G sodium salt, Streptomycin sulfate salt and Gentamicin sulfate salt were obtained from Sigma (St. Louis, MO). Restriction enzymes and peptide N-glycosidase (PNGaseF) were from New England Biolabs (NEB) (Revere, MA). Peroxidase-conjugated anti-rabbit antibodies and enhanced chemiluminescence (ECL) kit were obtained from GE Healthcare Amersham (Piscataway, NJ) and Invitrogen (Carlsbad, CA), respectively. SDS-polyacrylamide gel electrophoresis standards were purchased from Bio-Rad and the nitrocellulose membrane were from Schleicher and Schuell (Keene, NH). NHS-SS-Biotin and neutravidin beads were purchased from Thermo Fisher Scientific (Rockford, IL). mMESSAGE mMACHINE for *in vitro* transcription was obtained from Ambion (Austin, TX). ^3^H-labeled L-alanine was purchased from American Radiolabeled Chemicals (St. Louis, MO). Protease inhibitors were obtained from Roche Molecular Biochemicals (Mannheim, Germany). MTSEA-Biotin was purchased from Toronto Research Chemicals (Toronto, Canada). All other chemicals were either from Sigma (St. Louis, MO) or Fisher Scientific (Pittsburgh, PA).

### Construction of Mutants

Wild-type mouse SNAT4 in pcDNA3.1 vector was used to generate Cys mutants. Cys-null SNAT4, 4 cysteine to alanine mutants with only a single cysteine remaining; 18C, 232C, 249C, 321C and 345C, single site mutants; C18A, C232A, C249A, C321A, and C345A, and triple site mutant, C18A, C232A, C345A, were made using Quick Change Site Directed Mutagenesis Kit™ as per manufacturer’s instructions (Stratagene, La Jolla, CA). These DNA fragments were subcloned into a *Xenopus* oocyte expression vector, pGEMHE by digesting with *BamHI* and *ApaI* enzymes [23]. The identity of the mutants was confirmed by DNA sequencing (DNA Core Facility, UTHSCSA). The primers for PCR were designed to convert cysteine residue to alanine as listed in Table 1. All the plasmids were linearized using *AflIII* enzyme and *in vitro* transcribed by T7 RNA polymerase using mMessage mMachine kit (Ambion). cRNA was extracted and purified by lithium chloride and ethanol precipitation method according to the manufacturer’s instruction (Ambion). cRNA was resuspended in diethyl pyrocarbonated-treated water at a concentration of 1 µg/µl and stored at −80°C prior to use.

**Table 1:** Primer Sequences for site directed mutagenesis of cysteine residues.

MUTANTS	SENSE	ANTISENSE
**C18A**	ACGGAGACAGCTGCAGCGGGGACAGT	ACTGTCCCCGCTGCAGCTGTCTCCGT
**C232A**	GATTTTCTCTCTCCTGCATGGTGTTTTTCG	CGAAAAACACCATGCAGGAGAGAGAAAATC
**C249A**	ATTCCAAATTCCCTGCCCTCTGCCTG	CAGGCAGAGGGCAGGGAATTTGGAAT
**C321A**	GTGCTGAGAAATGCCAACCAAAATACT	AGTATTTTGGTTGGCATTTCTCAGCAC
**C345A**	CTTTTGCTTTTGTCGCCCACCCTGAGGT	ACCTCAGGGTGGGCGACAAAAGCAAAAG

### Transport Assays

*Xenopus laevis* oocytes were injected with 40 nl of cRNA of wild-type SNAT4 or Cys-null SNAT4 or mutants. Water injected oocytes were used as control. After 72 hr incubation at 17°C, the uptake assays were performed according to previously published procedure [24]. Oocytes were rinsed three times with the uptake buffer (KCl, 2 mM; MgCl_2_, 1 mM; CaCl_2_, 1 mM; HEPES, 10 mM; and Tris, 50 mM, 100 mM NaCl) and then incubated in the same buffer for 30 min at room temperature. Amino acid uptake was measured by incubating the oocytes with 500 µl of 50 µM [^3^H]-labeled L-alanine or [^3^H]-labeled L-arginine for 30 min at room temperature. Later, oocytes were washed three times with the same cold uptake buffer to terminate the uptake of L-alanine or L-arginine and were lysed with 1% SDS. The lysate was used for measurement of radioactivity with a scintillation counter in 5 ml scintillation solution. The results are presented as percentage of wild-type or untreated control and are expressed as the mean ± SEM. To study the effect of reducing agents on transport activities, the cRNA injected oocytes were incubated with appropriate amount of DTT [18], TCEP [25] or GSH for 30 min in modified Barth’s solution (MBS) (88 mM NaCl, 1 mM KCl, 2.4 mM NaHCO_3_, 0.82 mM MgSO_4_, 0.33 mM Ca(NO_3_)_2_, 0.41 mM CaCl_2_, 10 mM HEPES). The radioactive uptake solution was also prepared in MBS containing DTT, TCEP or GSH. The oocytes were then washed with ice cold MBS solution without the presence of the above mentioned reducing reagents. The results are presented as percentage of wild-type or untreated control after normalization with the level of SNAT4 protein and are expressed as the mean ± SEM.

### Preparation of Membrane Protein Extract

Crude membrane extracts were prepared from *Xenopus* oocytes. Oocytes were homogenized in lysis buffer (5 mM Tris, 5 mM EDTA and 5 mM EGTA at pH 8.0) containing protease inhibitors (NEM, PMSF, leupeptin and sodium vanadate). The homogenate was centrifuged at 6,600 g for 10 min at 4°C twice to remove the yolk and the supernatant was collected. The supernatant was then centrifuged at 100,000×g for 30 min at 4°C. The membrane pellet was dissolved in lysis buffer containing 1% SDS and the sample was loaded on 10% SDS/PAGE for western blot analysis.

### Cell Surface Biotinylation

Biotinylation of cells was performed based on the modification of previously published procedures [26]. Seventy-two hours after cRNA injection, 20 oocytes were labeled twice with 1 mg/ml Sulfo-NHS-LC-biotin at 4°C for 30 min each. The oocytes were then washed three times with PBS plus 100 mM glycine to stop the biotinylation reaction. In case of MTSEA-Biotin, 80 oocytes were labeled with 1 mM MTSEA-Biotin (prepared in ND100–100 mM NaCl, 80 mM mannitol, 2 mM KCl, 1.8 mM CaCl_2_, 1 mM MgCl_2_, 10 mM HEPES, and pH 7.5 adjusted with Tris-base) for 15 min at room temperature and washed three times with ND100 to stop the biotinylation reaction. Later, the oocytes were lysed and crude membrane protein extract was prepared as mentioned earlier. The pellet was dissolved in RIPA and TRIS buffer and incubated with streptavidin beads for overnight at 4°C. The beads were washed three times with phosphate buffered saline, and the biotinylated proteins were eluted by boiling for 5 min in a SDS-containing sample loading buffer. The total lysate and biotinylated samples (eluted from the streptavidin beads) were separated on SDS/PAGE and then immunoblotted with affinity-purified anti-SNAT4 antibody. The band intensities for the biotinylated and total protein were quantified using Scion Image software (Scion Inc.). Percentage of biotinylated (representing the surface pool) *versus* total (preloaded) SNAT4 was calculated. Data was analyzed by ANOVA followed by the Student-Newman-Keuls test to compare wild type and mutant SNAT4 biotinylated fractions. Data is presented as mean ± SEM.

### Western Blot Analysis

Rabbit anti-SNAT4 polyclonal antibody was produced as described previously [8]. The antisera generated were affinity-purified by passage through two Sepharose CL-4B columns, GST-conjugated and GST-SNAT4 fusion protein-conjugated, respectively. Membrane protein and biotinylation samples were loaded on 10% SDS/PAGE, transferred to the nitrocellulose membrane by semi-dry transfer apparatus (Bio-Rad) and the membrane was blocked with 10% non-fat dry milk for overnight. The membrane was probed with 1∶1000 dilution of affinity-purified anti-SNAT4 and followed by 1∶5000 dilution of peroxidase conjugated anti-rabbit secondary antibody. The antibody was detected using chemiluminescence reagent (ECL kit) according to manufacturer’s protocol. The membrane was then exposed to Phenix F-BX810 Blue X-Ray film and detected by autoradiography. Anti-pan-actin (Cell Signaling) antibody was used at 1∶1000 dilution and secondary antibody peroxidase conjugated anti-rabbit was used at 1∶5000 dilution.

## Results

### Disulfide Bond Reductants Decreased Transporter Activity of SNAT4

**Figure 1:**
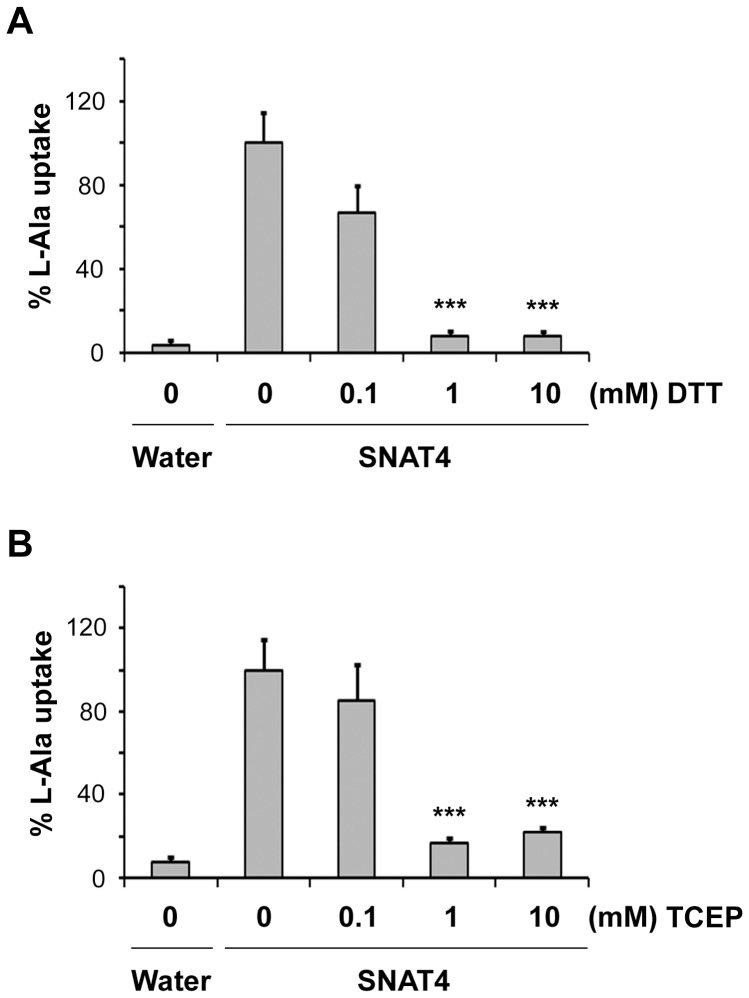
Dose-dependent Inhibition of SNAT4 Transport Activity by DTT and TCEP. Xenopus oocytes expressing wild type SNAT4 were preincubated with DTT (0–10 mM) (A) or TCEP (0–10 mM) (B) for 30 min. [^3^H] L-alanine uptake assay was then performed in the presence of DTT or TCEP. Water injected oocytes were used as a negative control. L-alanine uptake was significantly decreased in a dose-dependent manner compared to untreated control. Data is presented as mean ± SEM, n = 3 (∼ 10 oocytes/sample). DTT or TCEP at 1 and 10 mM versus untreated control of SNAT4, ***, P<0.001.

To determine the possible involvement of cysteine residues in the transport function of SNAT4 transporter, *Xenopus* oocytes injected with SNAT4 cRNA were treated with various concentrations of a membrane permeable reducing agent, DTT. [^3^H]-L-alanine uptake assay was performed. L-alanine transport in oocytes expressing SNAT4 decreased in a dose-dependent manner with increasing concentrations of DTT compared to untreated control (Fig. 1A). In the presence of both 1 mM and 10 mM DTT, the uptake was decreased drastically by 80%. To determine the membrane orientation of possible cysteine residue(s) involved, we used a membrane impermeable reducing reagent, TCEP. Treatment with TCEP also resulted in a dose-dependent loss of transport function of SNAT4 (Fig. 1B). These results indicate that cysteine residue(s) facing the extracellular side may play a role in transport function of SNAT4.

### Cysteine Residues are Important for Transport Activity, but not for Cell Surface Localization

**Figure 2:**
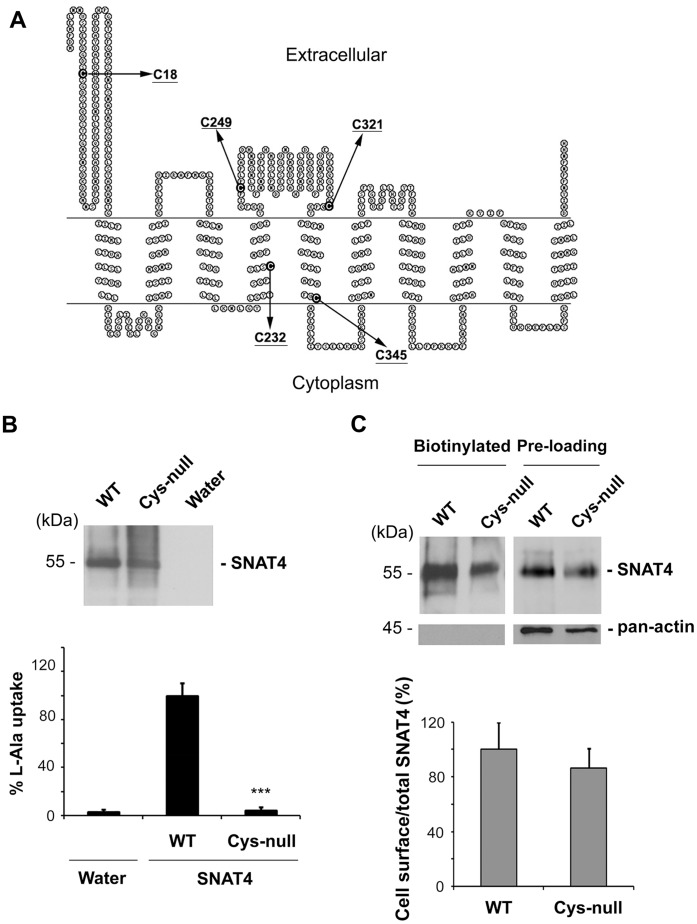
Cys-null mutant of SNAT4 completely loses transport function, but is capable of expressing on the cell surface. (A) The locations of the 5 cysteine residues are indicated (arrows) in the determined topological structure of SNAT4 [12]. (B) cRNA of the wild type and Cys-null mutant was injected into the oocytes and subjected to [^3^H] L-alanine uptake assays. Water injected oocytes were used as a negative control. The transporter activity obtained was normalized with the protein expression data. Data is presented as mean ± SEM, n = 3 (∼10 oocytes/sample). Cys-null versus WT, ***, P<0.001. (C) Xenopus oocytes injected with wild type and Cys-null mutant cRNA were surface biotinylated with NHS-SS-Biotin. Biotinylated proteins and the pre-loaded cell lysates (pre-loading) were also immunoblotted with anti-SNAT4 antibody or anti-pan-actin antibody. The ratio of biotinylated versus total SNAT4 was quantified and the data is presented as mean ± SEM, n = 20. The levels of SNAT4 expressed on the cell surface and corresponding pre-loaded SNAT4 were quantified by Scion Image software and the percentage of biotinylated versus total SNAT4 was calculated. Data is presented as mean ± SEM, n = 3 (∼ 20 oocytes/sample). All mutants versus WT, ***, P<0.001.

To identify cysteine residues essential for the transport function of SNAT4, site directed mutagenesis was performed to replace all 5 cysteine residues at positions of 18, 232, 249, 321 and 345 with alanine to generate a Cys-null mutant. The topological structure model of SNAT4 determined [12] shows three of the cysteine residues (C18, C249 and C321) to be extracellular and two (C232 and C345) to reside in the membrane (Fig. 2A). The cysteine mutants of SNAT4 used are listed in Table 2. The cRNA of the Cys-null SNAT4 or wild-type SNAT4 was injected into *Xenopus* oocytes and uptake assays were performed after 72 hours. In addition, the relative level of WT and mutant SNAT4 proteins expressed in oocytes was determined by western blots (Fig. 2B, upper panel). The transporter activity obtained was normalized to the level of SNAT4 protein expressed in the parallel cells. The result showed that Cys-null mutant of SNAT4 led to a complete loss of transporter activity (Fig. 2B), suggesting the essential role of cysteine residues in the substrate uptake function of SNAT4.

**Table 2:** Nomenclature of SNAT4 cysteine mutants.

MUTANT NAME	CHARACTERISTICS
Cys-Null	No cysteine remaining
18C; 232C; 249C; 321C; 345C	Single cysteine remaining/4 out of 5 cysteine mutated
C18A; C232A; C249A; C321A; C345A	Single site mutants/Single cysteine mutated to alanine
C18A, C232A, C345A	Mutant with retained disulfide forming cysteine residues

If the cysteines were involved in protein folding and trafficking, as is often observed, the inhibition of transport function by mutation of cysteines could be caused by the decrease of cell surface expression of SNAT4. *Xenopus* oocytes expressing wild-type and cysteine-null mutant SNAT4 were surface biotinylated with NHS-SS-biotin to assess the protein expression on the plasma membrane. The result showed similar levels of both total and surface expression between the cysteine-null and wild-type SNAT4 (Fig. 2C). The absence of intracellular pan-actin in biotinylated samples further validated the cell surface biotinylation assay. This data suggest that none of the five cysteine residues are required for delivery of the SNAT4 protein to the plasma membrane.

### Cysteine Residues, 249 and 321, Play an Important Role in the Transport Function of SNAT4

**Figure 3:**
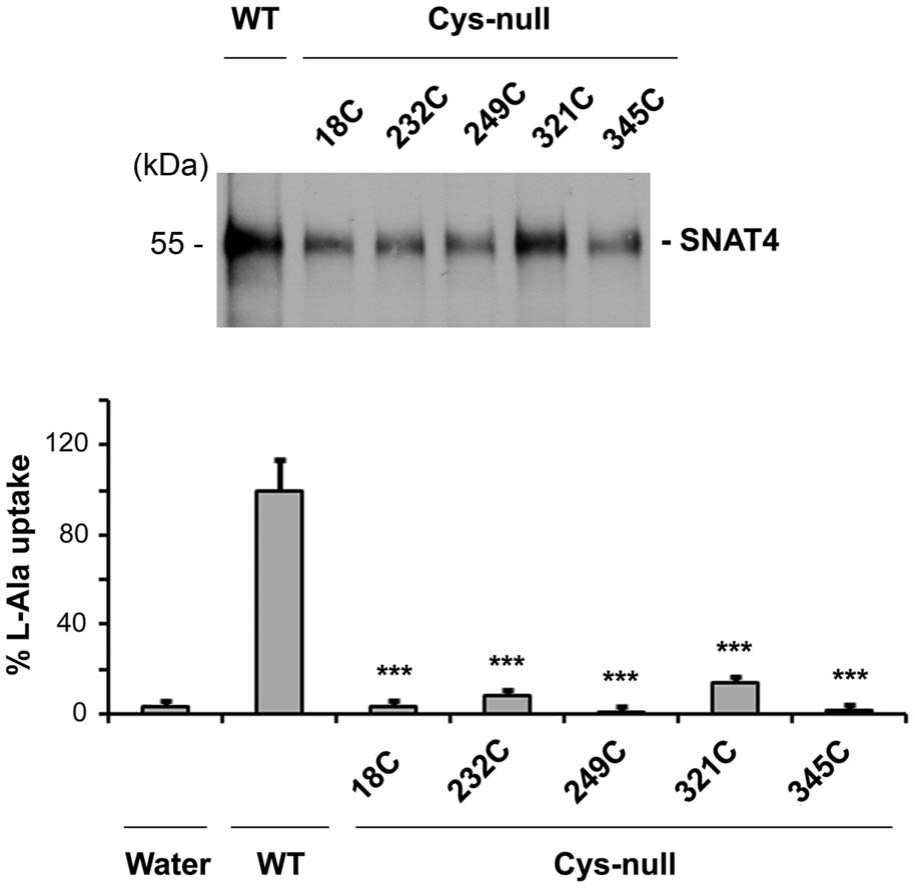
Replacing any four cysteines fails to recover transporter activity. DNA constructs containing 4 cysteine to alanine mutations with a single cysteine remaining, Cys-18 (18C), Cys-232(232C), Cys-249 (249C), Cys-321 (321C) or Cys-345 (345C) were generated by PCR using Cys-null SNAT4 as a DNA template. The cRNAs were injected in Xenopus oocytes. The transport activity was determined and the data was normalized with the SNAT4 protein level. Data is presented as mean ± SEM, n = 3 (∼10 oocytes/sample). All mutants versus WT, ***, P<0.001.

To identify the involvement of specific cysteine residue(s), we generated mutants (18C, 232C, 249C, 321C and 345C) by mutating 4 out of 5 cysteines to alanines with a single cysteine residue remaining. The expression level of WT and mutant SNAT4 protein expressed in oocytes was determined by western blots (Fig. 3, upper panel). As compared to wild-type SNAT4, all these five mutants showed a substantial loss of transporter activity (Fig. 3, lower panel). Since none of the single cysteine residues could restore the transporter activity of SNAT4, this result suggests that more than one cysteine residue may be involved in transport function of SNAT4.

**Figure 4:**
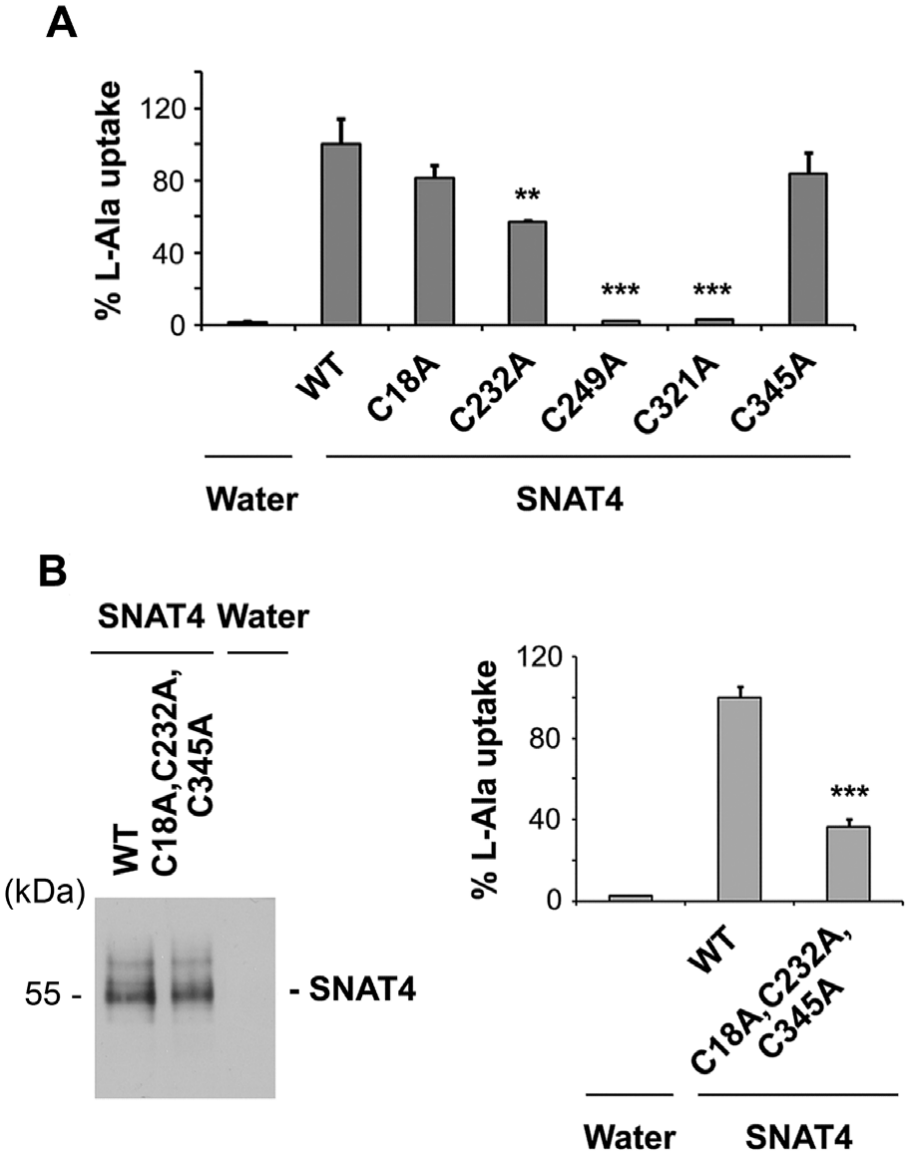
Residues Cys-249 and Cys-321 are functionally involved in transport function of SNAT4. (A) DNA constructs containing single cysteine site mutants, C18A, C232A, C249A, C321A and C345A were generated by PCR using WT SNAT4 as a template. cRNAs were injected in Xenopus oocytes. The transport activity was determined and the data was normalized with the protein expression. The transport activity of both C249A and C321A mutants was completely abolished. Data is presented as mean ± SEM, n = 3 (∼ 10 oocytes/sample). All mutants versus WT, ***, P<0.001. (B) DNA construct containing 3 cysteine to alanine mutations with retained Cys-249 and Cys-321 residues (C18A, C232A, C345A) was generated by site directed mutagenesis and the transporter activity was determined by uptake assay. After normalization with total protein expression of SNAT4 variants in oocyte (left panel), the mutant showed partial recovery in L-alanine transport as compared to the water injected control. Data is presented as mean ± SEM, n = 3 (∼10 oocytes/sample). The mutant versus WT, ***, P<0.001.

To identify the cysteine residues responsible for transporter activity, single cysteine to alanine mutants of SNAT4 namely, C18A, C232A, C249A, C321A and C345A, were generated. The transporter activity was determined by L-alanine uptake assay and the data was normalized with the level of SNAT4 protein (Fig. 4A). Mutants, C18A and C345A had no significant influence on alanine uptake compared to wild-type. Mutant C232A showed approximately 40% decrease in uptake function and mutation of C249A or C321A completely abolished the transport activity. This result suggests that C232A plays some role in transport, but cysteine residues 249 and 321 are essential for the substrate transport either individually or possibly by forming disulfide bond. To try to distinguish the latter, a mutant with three cysteines mutated to alanine (C18A, C232A, C345A) with only Cys-249 and Cys-321 residues remaining, was generated. *Xenopus* oocytes expressing this mutant SNAT4 had about 40% of L-alanine transport as compared to the oocytes expressing wild-type SNAT4 (Fig. 4B). This result was consistent with the observation that the C232A mutant reduced 40% of the activity of wild-type SNAT4 (Fig. 4A) and further confirmed that residues Cys-249 and Cys-321 are required for substrate transport function in SNAT4.

### Residues Cys-249 and Cys-321 are Linked by a Disulfide Bridge

**Figure 5:**
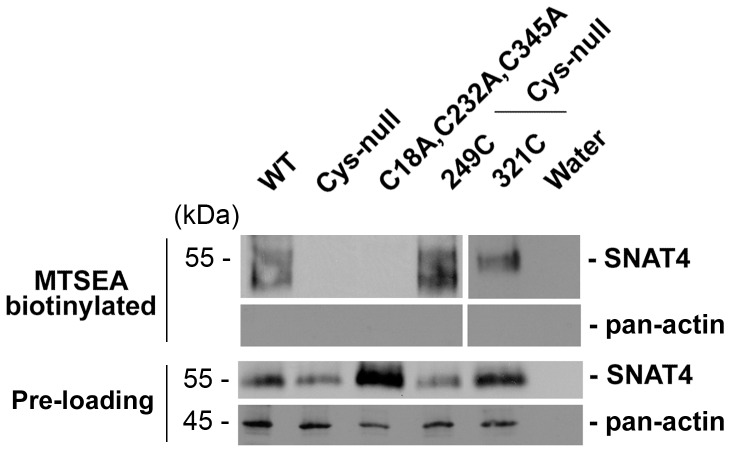
Residues Cys-249 and Cys-321 are linked by disulfide bridge. Xenopus oocytes injected with cRNAs of WT (lane 1), Cys-null mutant (lane 2), or mutants with retained 2 cysteine residues, Cys-249 and Cys-321 (C18A, C232A, C345A) (lane 3), 1 cysteine residue Cys-249 (249C) (lane 4) or 1 cysteine residue, Cys-321 (321C) (lane 5) were surface biotinylated by cysteine labeling with MTSEA-Biotin. Preloaded cell lysates (Pre-loading) and biotinylated samples were immunoblotted with anti-SNAT4 antibody or anti-pan-actin antibody (∼ 80 oocytes/sample).

To examine if residues Cys-249 and Cys-321 were directly linked by a disulfide bond in SNAT4, the membrane impermeable MTSEA-biotin reagent was employed. This reagent consists of a biotin group bound to the MTS derivative, MTSEA. MTSEA can form a disulfide linkage with a free thiol group of any exposed cysteine residue, resulting in labeling of the residue by biotin. However, the reagent does not react with oxidized disulfide bonds. Oocytes expressing wild-type SNAT4, or SNAT4 mutants containing intact Cys-249 and Cys-321, together (C18A, C232A, C345A) or alone (249C or 321C) were treated with MTSEA-biotin. The mutant with 249C or 321C was labeled by MTSEA-biotin (Fig. 5, lane 4 and lane 5). On the other hand, SNAT4 with both Cys-249 and Cys-321 (C18A, C232A, C345A) was not labeled by MTSEA-biotin (lane 3), suggesting that this mutant contained no free and accessible sulfhydryl groups. The results suggest that residues Cys-249 and Cys-321 are likely to form a disulfide bond in the SNAT4 transporter protein. Lack of intracellular pan-actin in the biotinylated samples suggests that MTSEA-biotin was only accessible to the cell surface proteins.

### Disruption of the Disulfide Bridge with Reducing Reagents Decreases Transport Function of SNAT4

**Figure 6:**
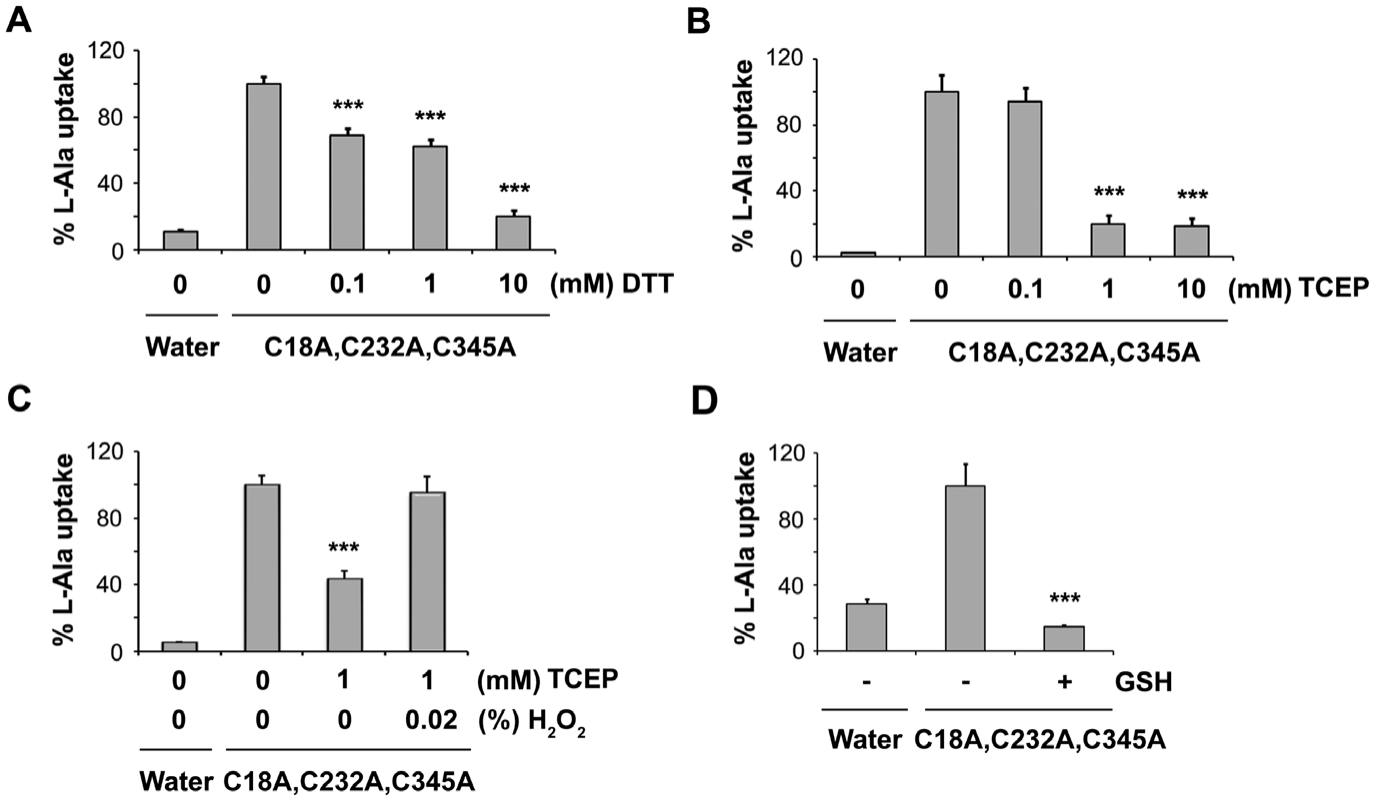
Transporter activity of cysteine mutant with only intact disulfide bridge (Cys-249 and Cys-321 residues) decreases in the presence of DTT, TCEP and GSH. Wild type and mutant SNAT4 expressing oocytes were incubated with DTT (0–10 mM) (A), TCEP (0–10 mM) (B) and 10 mM GSH (D). [^3^H]-alanine uptake assay was performed. Under reducing conditions, L-alanine transport in mutant SNAT4 significantly decreased. (C) Mutant SNAT4 expressing oocytes were incubated with 1 mM TCEP in the presence and absence of 0.02% H_2_O_2_. Under oxidative conditions, L-alanine transport in mutant SNAT4 was significantly recovered. Data is presented as mean ± SEM, n = 3 (∼ 10 oocytes/sample). Treated versus untreated control of the mutant, ***, P<0.001.

We then examined whether the disulfide bond plays a role in substrate transport by SNAT4. The uptake assay was performed with *Xenopus* oocytes expressing mutant retaining only two disulfide forming cysteine residues, Cys-249 and Cys-321 (C18A, C232A, C345A). In the presence of DTT, the L-alanine transport dramatically decreased in a dose-dependent manner as compared to the untreated control (Fig. 6A). A dose-dependent decrease in L-alanine uptake was also observed with increasing concentration of membrane impermeable TCEP (Fig. 6B). However, the inhibition in activity by reductant, TCEP was recovered under oxidative conditions in presence of 0.02% H_2_O_2_ (Fig. 6C). Furthermore, the oocytes expressing mutant SNAT4 were also treated with GSH, another membrane impermeable hydrosulfate reducing reagent. Consistent with the above results, treatment with GSH also showed a significant decrease in L-alanine uptake by SNAT4 mutant with only two disulfide bond-forming residues, Cys-249 and Cys-321 (Fig. 6D). These results clearly suggest that the disulfide bond formed between Cys-249 and Cys-321 is essential for the substrate transport by SNAT4.

### Disruption of the Disulfide Bridge Partially Impairs L-Arginine Transport of SNAT4

**Figure 7:**
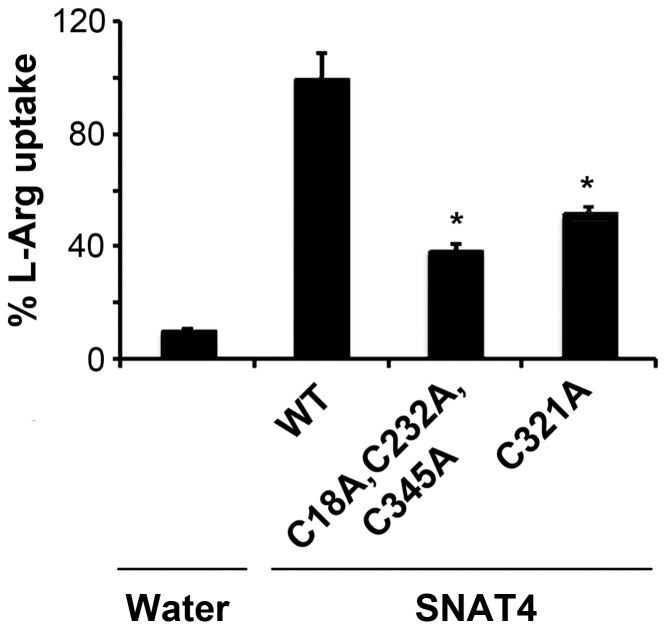
Disruption of the disulfide bridge partially loses L-arginine transport function of SNAT4. cRNA of the wild type, C18A, C232A, C345A or C321A mutant was injected into *Xenopus* oocytes and subjected to [^3^H] L-arginine uptake assays. Water injected oocytes were used as a negative control. Data is presented as mean ± SEM, n = 3 (∼10 oocytes/sample). Mutants versus WT, *, P<0.05.

In addition to L-alanine substrate, a previous report shows that SNAT4 is able to transport L-arginine [7]. We performed the similar transport assay in *Xenopus* oocytes using radioactive [^3^H]-L-arginine (Fig. 7). Consistent with the observation with L-alanine substrate, mutant retaining only the disulfide bridge (C18A, C232A, C345A) has about 40% of L-arginine transport function of wild-type SNAT4. Interestingly, the disruption of disulfide bridge (C321A) only lost about half of the activity, which is different from a complete loss for L-alanine substrate. This result suggests that the disulfide bridge appears to play a more prominent role in transport of neutral amino acids as compare to cationic amino acids. This is likely caused by the altered protein conformation due to the disruption of the disulfide bridge, which results in preferred transport of certain amino acid substrates over others.

## Discussion

In this study, we report, for the first time, the presence of a disulfide bond in a member of system N/A amino acid transporter SNAT family. There are four highly conserved cysteine residues, Cys-232, Cys-249, Cys-321 and Cys-345 across the members of SNAT family transporter proteins. Based on our recently resolved topological structure of the SNAT4 protein [12], the residues Cys-249 and Cys-321 reside on the third extracellular loop, whereas residues Cys-232 and Cys-345 are present on the fourth and fifth transmembrane domains, respectively. Our data suggests that residues Cys-232, Cys-249 and Cys-321 are involved in substrate transport of SNAT4. More importantly, the residues, Cys-249 and Cys-321, form a disulfide bond that is critical for the transport function by SNAT4. We further showed that cysteine residues and the identified disulfide bridge do not play a role in the cell surface expression of SNAT4.

The inter- and intra-disulfide bond formation is one of the major steps of protein modification process for proper protein folding and stability. Some disulfide bridges have been known to play a role in trafficking and function [13]–[15], whereas others regulate stability and oligomerization of transporters [16], [17], [19]. However, the formation of disulfide bonds has not yet been defined in any of the SNAT transporters. We showed the formation of an intra-molecular disulfide bridge between residues Cys-249 and Cys-321 located in the same extracellular loop domain of SNAT4. When we treated the samples under non-reducing conditions, we did not observe the presence of dimeric or multimeric forms of SNAT4. Therefore, it is unlikely that these two residues form intermolecular disulfide bond. Our initial evidence of the possible involvement of a disulfide bond in the SNAT4 transporter was that the substrate transport was strongly inhibited in the presence of a membrane-permeable reducing agent, DTT. The membrane impermeable reducing agent, TCEP also decreased the uptake parameters of wild-type SNAT4, implying the possible extracellular orientation of the disulfide bond(s). The involvement of cysteine residues was supported by the evidence that replacement of all five cysteines with alanine completely impairs the ability of SNAT4 to uptake L-alanine. Similar to SNAT4 transporter, the Cys-null mutant of GABA and OAT1 transporter proteins have also been reported to be non-functional [27], [28]. However, a fully functional Cys-null transporter has also been identified. Cys-null mutant generated in both proton-coupled folate transporter, PCFT-SLC46A1 [29] and glutamate transporter, GltT [30] have transporter activity similar to their wild-type. The basis for difference in the activity of the Cys-null mutants is not yet understood. To identify critical cysteines, we systematically removed 4 of 5 cysteine residues in all possible combinations. Disulfide bonds are formed by paired cysteine residues. Consistent with the requirement of a disulfide bond for SNAT4 function, we found no activity in any of the single mutants. We then tried the inverse strategy of eliminating only one cysteine at a time. These results indicate that Cys-18 and Cys-345 are not involved in the transport activity but that Cys-249 or Cys-321 likely are, as their deletion caused complete loss of L-alanine uptake. These residues, located on the third extracellular domain could function either alone or by formation of a disulfide bridge. The evidence that the mutant retaining only Cys-249 and Cys-321 had significant activity supports the possible existence of a disulfide bond formed by these two residues. The disulfide bridge is unlikely to be located at substrate binding site and thereby, may not be directly involved in substrate binding and translocation. However, the disulfide bridge could maintain protein structure integrity of the SNAT4 and thus the transport function of SNAT4. Additionally, mutation of transmembrane residue cysteine 232 to alanine significantly inhibited the transport function, suggesting that this residue may have a major influence on substrate transport of SNAT4.

The direct evidence for presence of the disulfide bond on the extracellular loop was obtained by using a membrane impermeable MTSEA-biotin reagent that reacts with non-disulfide bond forming cysteine residues. Mutating one of the two cysteine residues involved in formation of the disulfide bonds would leave an accessible “free” cysteine residue. While the mutant retaining only Cys-249 or Cys-321 did react with MTSEA-biotin, mutants with both Cys-249 and Cys-321 residues failed to react with MTSEA-biotin, consistent with their involvement in a disulfide bond. Since the residues 249 and 321 are conserved across animal species (human, mouse, rat and pongo) and among the members of the SNAT family, it is likely that the disulfide bond is also a conserved structural feature among the members of the SNAT family transporters. Interestingly, both the disulfide bridge-forming cysteines are present on the large extracellular loop domain, which appears to be a conserved structural feature in other transporters, e.g. GABA transporters, glutamate transporters [31], [32]. Therefore, there is a high probability of these conserved cysteines in formation of a disulfide bridge in SNAT and possibly in other transporters as well.

We also observed that 5 cysteine residues and the formation of disulfide bond are not essential for delivery of SNAT4 to plasma membrane. This part is contradictory with conventional dogma that formation of proper disulfide bonds is critical for protein folding and transport to plasma membrane, a classical “quality control” mechanism for secretory pathway. Previous reports have shown that disruption of disulfide bonds leads to protein retention and degradation in transporters, such as sodium phosphate cotransporter [25], dopamine transporter [14] and membrane proteins, such as CD36 [33] and ABC transporters ABCB6 and sulfonylurea receptor 1 [34]. The study in sodium phosphate cotransporter showed that formation of at least one disulfide bridge is essential to allow surface expression of functional transporter. On the contrary but consistent with our observation, another study reported that disruption of disulfide bridge of human proton-coupled amino acid transporter, hPAT1 does not affect the surface expression but abolishes the transport function [15]. Furthermore, studies in GABA, GAT, DAT and SERT transporters have identified phosphorylation as one of the key regulatory factors in trafficking of transporters to membrane. Signaling molecules, such as PKC and tyrosine kinases have been reported to play a role in regulation of cell surface expression of transporters (reviewed by [35]). Interestingly, a recent study showed that rat glutamine transporter SNAT3 is also post-translationally modified by phosphorylation [36]. This study showed that the transporter is phosphorylated at the serine residue (S52) in the N-terminus and is responsible for sequestration of the protein in the intracellular reservoirs. Taking into consideration the above reports, it is possible that membrane localization of SNAT4 could be regulated by other post-translational modifications such as phosphorylation. The disulfide bond of SNAT4 is likely to be directly involved in stabilizing the three-dimensional structure or the translocation pore of the protein, but also for the conformational changes that SNAT4 would undergo during the transport cycle. Advanced structural studies will be undertaken to delineate the importance of the disulfide bridge in SNAT transporters.
