# Correction: Identification of a disulfide bridge important for transport function of SNAT4 neutral amino acid transporter

**DOI:** 10.1371/journal.pone.0329196

**Published:** 2025-07-31

**Authors:** Rugmani Padmanabhan Iyer, Sumin Gu, Bruce J. Nicholson, Jean X. Jiang

After publication of this article [1], concerns were raised about Fig 5.

**Fig 5:**
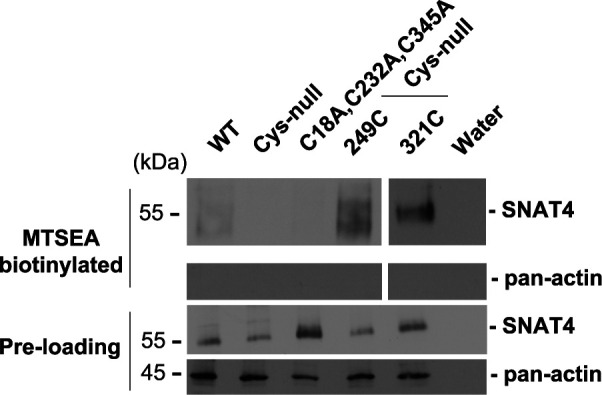
Residues Cys-249 and Cys-321 are linked by disulfide bridge. Xenopus oocytes injected with cRNAs of WT (lane 1), Cys-null mutant (lane 2), or mutants with retained 2 cysteine residues, Cys-249 and Cys-321 (C18A, C232A, C345A) (lane 3), 1 cysteine residue Cys-249 (249C) (lane 4) or 1 cysteine residue, Cys-321 (321C) (lane 5) were surface biotinylated by cysteine labeling with MTSEA-Biotin. Preloaded cell lysates (Pre-loading) and biotinylated samples were immunoblotted with anti-SNAT4 antibody or anti-pan-actin antibody (∼ 80 oocytes/sample).

Specifically:

- When levels are adjusted to visualize the background in the Fig 5 MTSEA biotinylated - SNAT4 panel, lanes 2 and 3 contain patterns in the background that appear similar and there appears to be a vertical discontinuity between lanes 2 and 3.
- The preparation of Fig 5 did not adhere to journal requirements. The corresponding author noted that:
  - The left-hand MTSEA biotinylated - SNAT4 panel is a composite image originating from two different exposures of the same blot, with lane 4 representing a lower exposure.
  - Spacing between the lanes of the Preloading - SNAT4 panel was adjusted to align with the rest of the figure, but image splicing was not clearly marked on the figure.

Here, the authors provide the original images underlying all panels in Fig 5 (S1 File) and repeat data for lanes 1–4 of the Fig 5 MTSEA biotinylated - SNAT4 panel, stated to be completed at an earlier date (S2 File). The corresponding author stated that the underlying data is available for all figures.

Regarding the similarity observed between lanes 2 and 3 in the MTSEA biotinylated - SNAT4 panel of Fig 5, the first author stated lane 1 originates from the original blot following a longer exposure, and lanes 2–4 originate from the same blot with a shorter exposure. Editorial assessment by PLOS determined that lanes 2 and 3 in the published MTSEA biotinylated - SNAT4 panel of Fig 5 do not appear to originate from the underlying image provided, which appears to show faint signals in lanes 2 and 3, but that both the original and repeat blots provided for this panel provide support for the negative results presented in these lanes. PLOS consulted a member of the *PLOS One* Editorial Board who indicated the presence of faint signal in lanes 2–3 does not affect the reported conclusions. They noted the limitations for this experiment that pan-actin controls and the 321C sample were run on separate gels and that the molecular weight marker information is not clear in the underlying image for 321C, but advised that the overall conclusions, which are supported by other data in the article, are reasonable and well-founded.

With this Correction, the first author provides a revised Fig 5 with the MTSEA biotinylated - SNAT4 lanes 1–4 and Preloading - SNAT4 panels replaced with the original, unedited panels.

## Supporting information

S1 File Original image data underlying Fig 5. This file includes the original images underlying the 6 panels in Fig 5: MTSEA biotinylated - SNAT4, lanes 1–4; MTSEA biotinylated - SNAT4, lanes 5–6; MTSEA biotinylated – pan-actin, lanes 1–4; MTSEA biotinylated – pan-actin, lanes 5–6; Preloading - SNAT4; Preloading – pan-actin.
(ZIP)

S2 File Image data underlying repeat data for Fig 5. This file includes repeat data for Fig 5 MTSEA biotinylated - SNAT4, lanes 1–4.
(ZIP)
