# Supplementary figures and images for: Correction: Identification of a disulfide bridge important for transport function of SNAT4 neutral amino acid transporter
Source: PLoS One. 2025 Jul 31;20(7):e0329196. doi: 10.1371/journal.pone.0329196 (PMC12312896; doi:10.1371/journal.pone.0329196)

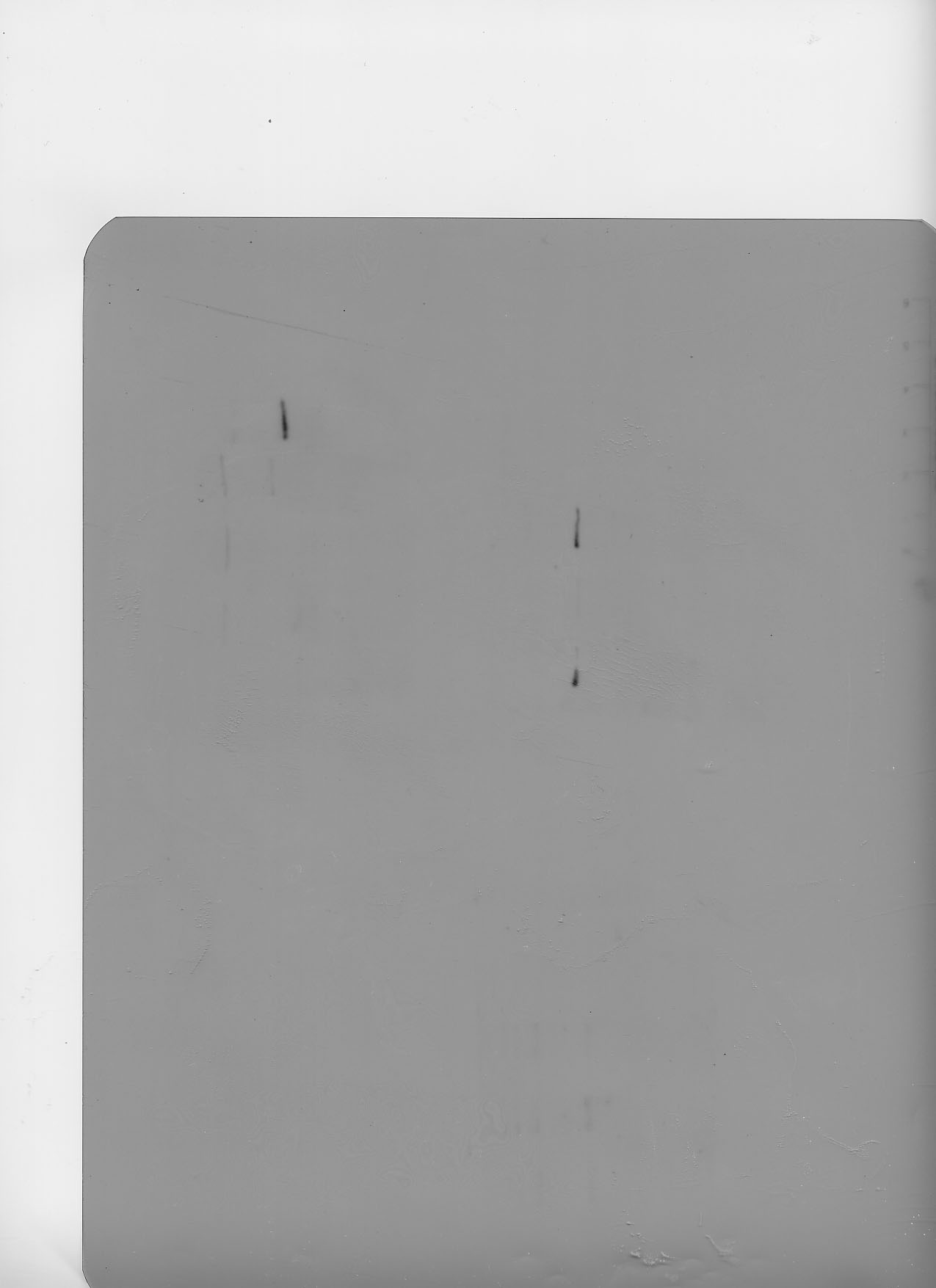

Supplement: S1 File — Original image data underlying Fig 5. This file includes the original images underlying the 6 panels in Fig 5: MTSEA biotinylated - SNAT4, lanes 1–4; MTSEA biotinylated - SNAT4, lanes 5–6; MTSEA biotinylated – pan-actin, lanes 1–4; MTSEA biotinylated – pan-actin, lanes 5–6; Preloading - SNAT4; Preloading – pan-actin. (ZIP) [file pone.0329196.s001.zip › S1 File/Fig 5. MTSEA biotinylated - pan-actin, lanes 5-6.jpg]

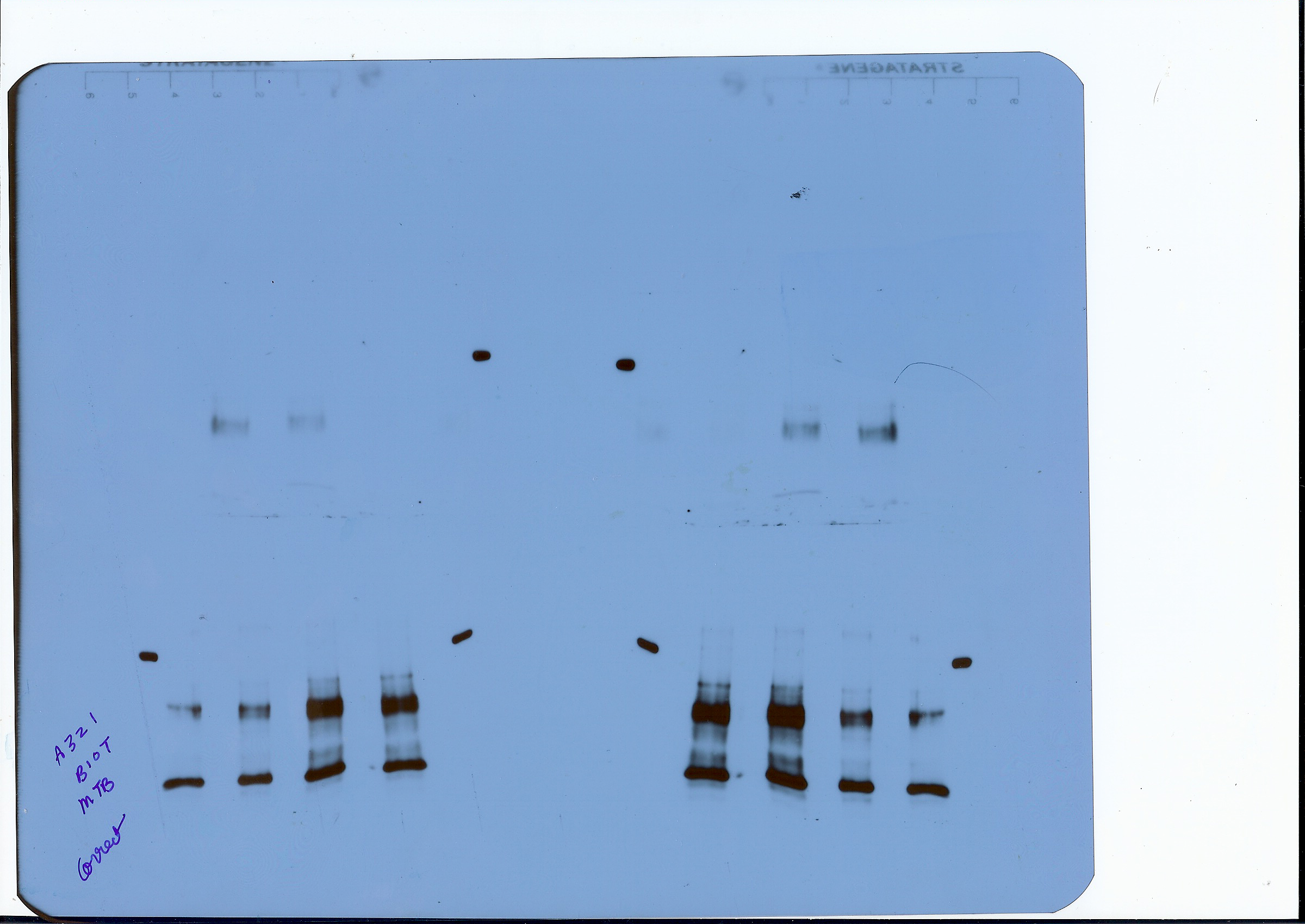

Supplement: S1 File — Original image data underlying Fig 5. This file includes the original images underlying the 6 panels in Fig 5: MTSEA biotinylated - SNAT4, lanes 1–4; MTSEA biotinylated - SNAT4, lanes 5–6; MTSEA biotinylated – pan-actin, lanes 1–4; MTSEA biotinylated – pan-actin, lanes 5–6; Preloading - SNAT4; Preloading – pan-actin. (ZIP) [file pone.0329196.s001.zip › S1 File/Fig 5. MTSEA biotinylated - SNAT4, lanes 5-6.tif]

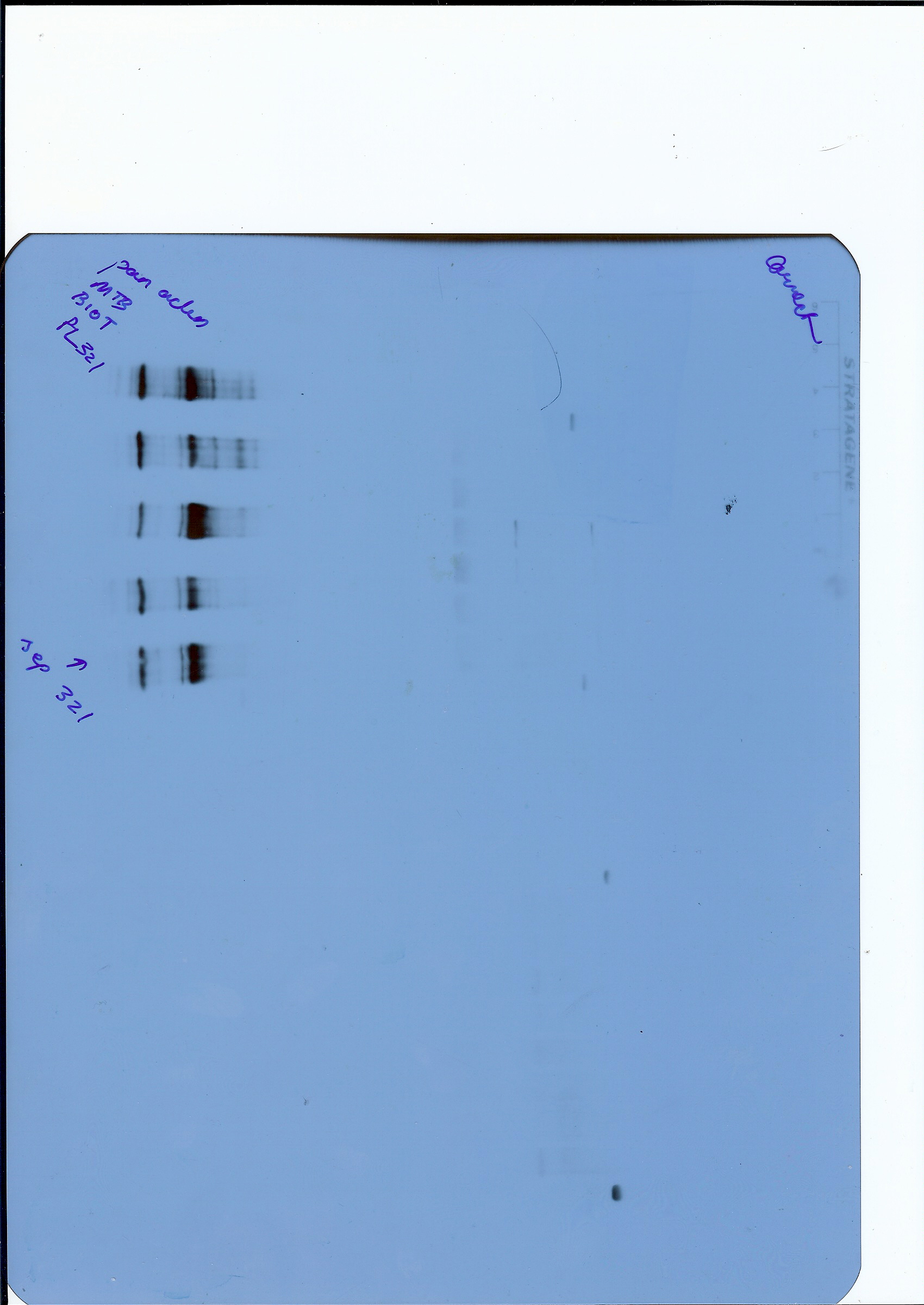

Supplement: S1 File — Original image data underlying Fig 5. This file includes the original images underlying the 6 panels in Fig 5: MTSEA biotinylated - SNAT4, lanes 1–4; MTSEA biotinylated - SNAT4, lanes 5–6; MTSEA biotinylated – pan-actin, lanes 1–4; MTSEA biotinylated – pan-actin, lanes 5–6; Preloading - SNAT4; Preloading – pan-actin. (ZIP) [file pone.0329196.s001.zip › S1 File/Fig 5. Preloading - pan-actin.jpg]
